# Quantifying Post- Laser Ablation Prostate Therapy Changes on MRI via a Domain-Specific Biomechanical Model: Preliminary Findings

**DOI:** 10.1371/journal.pone.0150016

**Published:** 2016-04-18

**Authors:** Robert Toth, Dan Sperling, Anant Madabhushi

**Affiliations:** 1 Department of Biomedical Engineering, Case Western Reserve University, Cleveland, OH, United States of America; 2 Toth Technology LLC, Long Valley, NJ, United States of America; 3 Sperling Prostate Center, Manhattan, NY, United States of America; University of Minnesota, UNITED STATES

## Abstract

Focal laser ablation destroys cancerous cells via thermal destruction of tissue by a laser. Heat is absorbed, causing thermal necrosis of the target region. It combines the aggressive benefits of radiation treatment (destroying cancer cells) without the harmful side effects (due to its precise localization). MRI is typically used pre-treatment to determine the targeted area, and post-treatment to determine efficacy by detecting necrotic tissue, or tumor recurrence. However, no system exists to quantitatively evaluate the post-treatment effects on the morphology and structure via MRI. To quantify these changes, the pre- and post-treatment MR images must first be spatially aligned. The goal is to quantify (a) laser-induced shape-based changes, and (b) changes in MRI parameters post-treatment. The shape-based changes may be correlated with treatment efficacy, and the quantitative effects of laser treatment over time is currently poorly understood. This work attempts to model changes in gland morphology following laser treatment due to (1) patient alignment, (2) changes due to surrounding organs such as the bladder and rectum, and (3) changes due to the treatment itself. To isolate the treatment-induced shape-based changes, the changes from (1) and (2) are first modeled and removed using a finite element model (FEM). A FEM models the physical properties of tissue. The use of a physical biomechanical model is important since a stated goal of this work is to determine the physical shape-based changes to the prostate from the treatment, and therefore only physical real deformations are to be allowed. A second FEM is then used to isolate the physical, shape-based, treatment-induced changes. We applied and evaluated our model in capturing the laser induced changes to the prostate morphology on eight patients with 3.0 Tesla, T2-weighted MRI, acquired approximately six months following treatment. Our results suggest the laser treatment causes a decrease in prostate volume, which appears to manifest predominantly at the site of ablation. After spatially aligning the images, changes to MRI intensity values are clearly visible at the site of ablation. Our results suggest that our new methodology is able to capture and quantify the degree of laser-induced changes to the prostate. The quantitative measurements reflecting of the deformation changes can be used to track treatment response over time.

## Introduction

### Background and Motivation

Following a diagnosis of prostate cancer, several treatment options are available. These include brachytherapy, focal laser ablation therapy, hormonal therapy, external beam radiation therapy, and radical prostatectomy. Over 90% of low risk prostate cancer is currently treated with radical treatment [1], which causes significant quality of life issues and side effects such as incontinence, impotence, and damage to surrounding organs [2–4]. One alternative to radical treatment is active surveillance, which involves actively monitoring disease related changes to assess whether or not treatment should be performed, in order to mitigate the quality of life issues associated with radical treatment.

Focal laser ablation has recently emerged as an extremely promising prostate cancer treatment since it includes the best attributes of radical treatment (the ability to eradicate cancer cells), and its precision allows one to minimize the risk of side effects [1–6]. Focal laser ablation causes thermal destruction of tissue by a laser [1]. Radiation from a laser is absorbed by the tissue, causing homogeneous thermal necrosis [2]. Due to the lack of excess vascularity in the prostate (which could cause unwanted excess conduction of heat), prostate cancer is well-suited for focal laser ablation treatment [6]. Focal laser ablation for prostate cancer has the additional advantages of ease of use, and lower cost than some radical treatments [1].

However, since focal laser ablation is such a new technology, few studies have looked at the long term effects of such treatment for prostate cancer. It is possible that studying early treatment changes on imaging may help to identify markers associated with longer term prognosis [7]. As such, a quantitative method for systematically and quantitatively tracking treatment related changes over time could potentially be used to predict longer term outcome.

MRI is used to both guide the focal laser ablation treatment, and to evaluate its efficacy [2, 4–7]. Prior to treatment, MRI is used to locate the tumor or index lesion [2, 6], and is used to guide the laser during treatment [2]. Following focal laser ablation, MRI can be used to determine the effect of ablation [5],[7], calculate the size of the ablated lesion [5], detect cancerous tissue [2, 4], and detect complications with surrounding organs such as the rectum or neurovascular bundle [4]. Raz et al. observed that a contrast-enhanced MRI directly following treatment can be used to confirm the treatment success, or immediately repeat the focal laser ablation during the same session [4]. Only seven days following treatment, hypoperfused lesions (lesions with decreased blood flow) were evident on MRI [3]. Eggener et al. recommended that following focal laser ablation, in addition to biopsies, periodic MRI should be performed in order to characterize treatment effects [3].

Assuming that MRI can successfully be used to determine treatment efficacy [2, 4–7], it stands to reason that quantitatively determining changes on MRI following treatment can be used to systematically track the effects of treatment over time. These quantitative changes on the prostate can manifest as either shape-based or functional changes in the prostate. Shape-based changes are useful in order to determine how the volume of the tumor changes following treatment, and how those changes affect the size and shape of the prostate gland as a whole. Functional changes are useful in order to determine how focal laser ablation changes the tissue properties within the prostate. While T2-weighted MRI intensity values are the result of several underlying physical processes, it is our assumption that functional changes can be characterized by changes in the intensity values and texture on the MRI in the ablated region. These changes in appearance over time may be due to necrosis of healthy tissue from the laser, or from destruction and elimination of tumor cells from the ablation. Our previous study [7] explored the correlations which may exist between MRI intensity features and functional changes in the prostate. This work aims to compare pre- and post-treatment MRI in order to quantify (a) functional and (b) shape-based changes to the prostate due to the ablation.

Previous work on quantitatively evaluating post-ablation effects on the prostate via MRI [7] involved considering how different MR parameters such as apparent diffusion coefficient change following ablation. What separates this work from previous work [7] is (1) a larger cohort size, (2) the use of a deformable, non-linear, biomechanically constrained registration technique to align the pre-, post-treatment imagery, and (3) the removal of the confounding effects from the bladder and rectum.

In this study, it is assumed that the interventional radiologist outlines the tumor region and ablation zone prior to focal laser ablation, as well as the prostate, bladder, and rectum on each image. In order to determine the effects of focal laser ablation following treatment, the ablated zone must first be identified on the post-treatment MRI. However, a direct spatial mapping is not possible on the follow-up MRI due to:

1. Differences in patient position within the MRI machine.
2. Changes in the prostate due to motion and filling of nearby tissue and organs such as the bladder and rectum.
3. Shape-based changes in the prostate due to the ablation.

As such, a registration algorithm is required to address these changes. The first problem enumerated above can be addressed by a linear (rigid or affine) alignment of the pre-, post-treatment MRI. The second challenge can be addressed by a non-linear (deformable) model specifically designed to simulate the changes to the prostate due to nearby tissues. The third challenge can be addressed by a non-linear alignment of the pre-, post-treatment prostate surfaces. In fact, as stated previously, an exploration of the third challenge (the shape-based changes due to focal laser ablation) is one of the stated goals of this work.

In order to isolate the shape-based changes due primarily to the ablation induced necrosis, changes in shape due to the motion of nearby organs must be excluded. If the motion due to external forces from nearby organs is completely removed, then the only remaining changes in morphology will be due to the ablation itself. Since this work aims to quantitatively track the changes in morphology from the ablation treatment, the motion of the nearby organs must be taken into account separately. This will allow us to study the deformations induced primarily by the focal laser ablation. While additional confounding factors (such as blood flow) may to the prostate deformation in the period between treatment and followup imaging, in this study we assume that the dominant induced deformation is primarily due to the largest surrounding organs (bladder and rectum) and the treatment itself.

A finite element model (FEM) is a useful tool in order to model the biomechanical changes the prostate undergoes, which this work aims to interrogate. A FEM is a biomechanical model which uses physical properties such as elasticity and compressibility to deform one or more objects. It is a popular method for modeling physical deformations due to its ability to constrain the resulting deformation to only biomechanically real changes based on physical tissue properties. Another advantage of using a FEM to model these changes is that FEM’s are well studied in mechanical engineering, and thus provide a standardized biomechanical registration method for tracking treatment related changes over time.

Brock et al. [8] developed a technique for non-linearly aligning the surfaces of two prostates using a FEM. In [9] we employed a FEM to non-linearly align pre-, post-treatment prostate T2-w MRI by simulating the morphological shrinking effects of external beam radiation treatment. However, in our previous approach, the morphological effects of the treatment were known *a-priori*, and the goal was simply to spatially align the pre- and post-treatment prostate MRI scans. By contrast, in this approach we wish to explore the morphological effects of the focal laser ablation treatment by determining changes in the prostate shape post-treatment. As such, as stated previously we attempt to explicitly isolate only the focal laser ablation induced changes to the prostate. This entails eliminating the deformations from patient motion as well as other, non-treatment related forces, such as the motion of nearby organs.

The overarching goals for this work are firstly to quantify changes in prostate volume post-treatment. Secondly, we will isolate and quantify treatment-induced shape-based changes. Both changes in gross volume as well as localized treatment-induced morphology changes will allow us to understand how laser treatment affects the gland morphology, shape, and size, and may be useful in predicting long term patient outcome. Thirdly, we will quantify differences in MRI intensity values pre-, post-treatment. Changes in MRI parameters following treatment may be useful in determining treatment related effects such as edema and necrosis [7]. As such, predictive models may be generated in order to use early changes in MRI intensity values to predict long term patient outcome. In addition, quantifying these changes will allow clinicians to have a system for quantitatively tracking treatment response over time.

### Previous Work and Methodological Contributions

**Fig 1:**
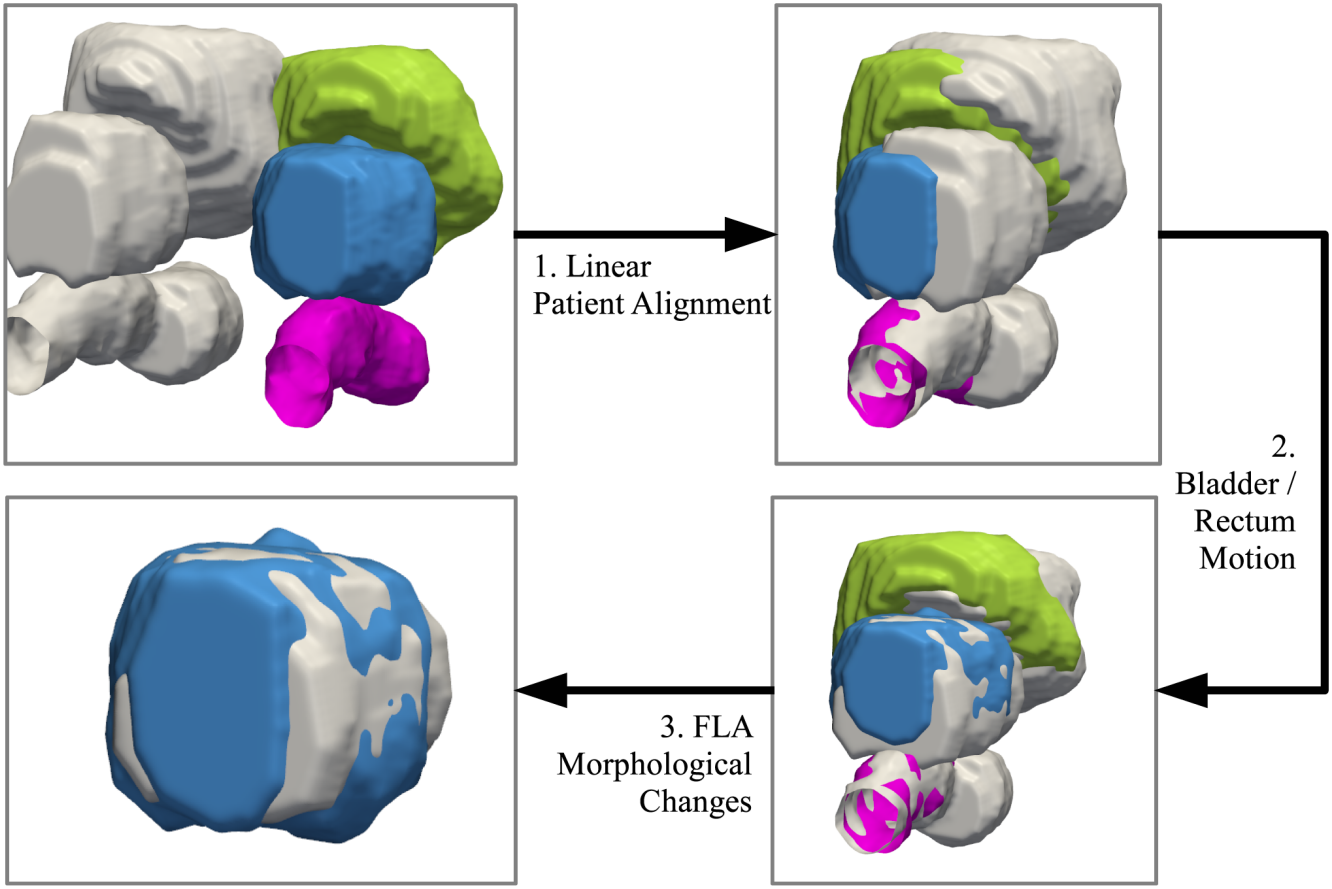
Overview of the registration techniques used to bring the pre-, post-treatment into spatial alignment. The post-treatment bladder, prostate, and rectum are shown in green, blue, and pink, respectively. The pre-treatment surfaces are shown in grey. The first step is to use a linear registration to account for patient alignment. Secondly, a finite element model (FEM) is used to calculate the deformation due to the bladder and rectum, and removes those deformations. The preceding two steps are necessary in order to remove confounding deformations and isolate the treatment-induced changes to the gland. Finally, a second FEM calculates the remaining deformations on the prostate. These deformations can therefore be assumed to be primarily (if not exclusively) due to shape-based changes from the ablation. The quantitative measurements reflecting such changes can be used to track treatment response over time.

An overview of the registration steps for bringing the pre-, post-treatment scans into spatial alignment is shown in Fig 1. To model the deformations due to the patient alignment, a linear registration technique is performed in which translation, rotation, and scaling are used to optimally align the pre-, post-treatment. To model the motion of the bladder and rectum, as well as the shape-based changes due primarily to the focal laser ablation, a finite element model (FEM) is used.

In [10], outlines of the tumor, prostate, and its substructures were manually identified, and subsequently manually brought into alignment to evaluate early treatment response of radiotherapy for prostate cancer by studying changes in the apparent diffusion coefficient on MRI following spatial alignment of the scans. In our approach, the bladder, rectum, and internal structures of the prostate are outlined on both pre- and post-treatment MRI, and a biomechanical model is used to automatically register the images.

An FEM deforms a model of an organ based on physical properties of the organ, specifically Young’s modulus and Poisson’s ratio. Young’s modulus defines the “hardness” of the tissue. In this work we use the Young’s modulus to define the degree to which a force applied to the prostate will deform the tissue.

There are several examples of biomechanical models being used to register prostate images [8, 11–15]. In addition, there are several examples of biomechanical models being used to register pre-, post-treatment imagery [16–18]. Yet to the best of our knowledge, this is the first time a biomechanical model has been used to specifically register prostate focal laser ablation pre-, post-treatment imagery.

Existing prostate biomechanical models have focused on how external loads on the surface of the prostate deform the gland [8, 11–15], which is extremely useful when modeling how adjacent organs move relative to each other [11, 14], or how an object deforms that prostate [13]. Chi et al. [11] modeled how well FEMs captured the motion of the bladder, prostate, and rectum on CT imagery, and explored different material properties for benign prostate tissue, prostate tumors, and benign prostatic hyperplasia. Boubaker et al. [14] used a FEM to model how the bladder, rectum, and prostate moved on CT imagery, and compared the results to a cadaver. Crouch et al. [12] used a FEM to register the prostate surfaces on CT imagery. Hensel et al. [13] used a FEM to register MRI with an endorectal coil to MRI without. Brock et al. [8] performed MRI-to-MRI registration by automatically aligning nodes on the surface of the prostate.

In this work, we will generate a FEM to determine how the motion and filling of the bladder and rectum affect the prostate. This simulated motion will be inverted, so that the only remaining changes in the prostate are due primarily to the focal laser ablation. A second FEM will then be used to model the shape-based changes in the prostate due primarily to the focal laser ablation. This will allow us to (1) determine the changes to the MRI parameters specifically at the ablated zone, and (2) determine the shape-based changes induced by focal laser ablation to the prostate and its internal structures.

Our approach provides the basis for isolating treatment-related shape-based changes to the given tissue, which is also applicable for isolating treatment-related shape-based changes for different types of focal treatments for prostate cancer such as brachytherapy and radiofrequency ablation. In addition, it is also applicable to isolating treatment-related effects in other organs and diseases, such as lung tumors and liver cancer [19, 20]. Removing the effects of nearby organ motion (such as the heart for lung tumors) is critical for determining treatment efficacy [19, 20].

## Methods

### Notation and Preliminaries

A 3D MRI scene *I* = (*C*, *f*(*c*)) is defined by a collection of voxels *c* = (*x*_*c*_, *y*_*c*_, *z*_*c*_), ∀*c* ∈ *C*, and MRI intensity information for each voxel, f(c)∈R, ∀*c* ∈ *C*. The pre-treatment MRI is denoted as *I*_*Pre*_ and the post-treatment MRI is denoted as *I*_*Post*_. An image scene deformed by transformation *T* is defined as,

$$\begin{array}{c} T(I)=(C,f(T(c))), \end{array}$$ (1)

where *T*(*c*) represents the transformation of voxel *c*. *T*(*C*) represents the collection of transformed voxels, *T*(*C*) = {*T*(*c*) ∣ ∀*c* ∈ *C*}.

Following treatment, we assume the prostate undergoes transformations due to different patient alignment within the MRI machine (*T*_1_), transformations due to changes in surrounding tissue (*T*_2_), and focal laser ablation-induced transformations (*T*_3_). Therefore,

$$\begin{array}{c} C_{Post}=T_{1}\left(T_{2}\left(T_{3}\left(C_{Pre}\right)\right)\right). \end{array}$$ (2)

It follows that,

$$\begin{array}{c} T_{3}\left(C_{Pre}\right)={\hat{T}}_{2}\left({\hat{T}}_{1}\left(C_{Post}\right)\right), \end{array}$$ (3)

where $\hat{T}\left(\cdot \right)$ represents the inverse transformation *T*^−1^(⋅).

### Methdological Overview

The following sections outline the procedure for calculating the inverse transformations ${\hat{T}}_{1}$, ${\hat{T}}_{2}$. Calculating the deformation due to an external object on the prostate, and applying the inverse of such deformation to the prostate surface, has the effect of essentially reversing, or subtracting, that external deformation from the shape of the prostate. Any differences in prostate shape which remain between the pre- and post-treatment surfaces (calculated as *T*_3_) (after subtracting the deformations due to external objects) likely stem primarily from the effects of ablation.

One of the stated goals of this work is to isolate these focal laser ablation induced shape-based changes *T*_3_. In addition, once ${\hat{T}}_{1}$, ${\hat{T}}_{2}$, and *T*_3_ are known, the MRI parameters can be compared between *T*_3_(*I*_*Pre*_) and ${\hat{T}}_{1}\left({\hat{T}}_{2}\left(I_{Post}\right)\right)$, which represents spatially aligned, pre-, post-treatment MRI.

### Linear Alignment ${\hat{T}}_{1}$ of Pre-, Post-Treatment Scans

The first step in accounting for the focal laser ablation induced deformation is to linearly align the pre-, post-treatment MRI. This will presumably remove the effects of different patient positioning within the scanner and different field of views of the pre-, post-treatment scans. A linear transform is defined by translation, rotation, and scaling, in each of the three dimensions. The mutual information (*MI*) between the pre-, post- MRI is used as the metric to guide the linear registration. A gradient descent optimizer is used to determine which transformation yields the maximum mutual information, defined as,

$$\begin{array}{c} {\hat{T}}_{1}=\arg\max_{T}\;MI\left(T\left(I_{Post}\right),I_{Pre}\right). \end{array}$$ (4)

### Simulating Deformations on an MRI Scan due to External Physical Forces via Finite Element Modeling

An FEM contains elements (e.g. hexahedrons) connected at nodes. This section describes how the FEM incorporates external forces to deform an MR image C.

The FEM was constructed by dividing the manual segmentation of the prostate, bladder, and rectum, into hexahedron elements [12, 21]. The size of the elements used was dependent on the curvature of the model in that region, such that locations with higher curvature (e.g. near the edges of the 3D segmentation) were subdivided into smaller hexahedrons to account for minute changes, compared to the regions within the prostate (to account for gross changes).

Given *N* nodes N in a 3D FEM, a 3*N* × 3*N* sparse, symmetric “stiffness” matrix *K* defines how each node interacts with every other node. A 3*N* × 1 vector *V* represents the coordinates of the nodes, a 3*N* × 1 vector *F* represents a series of external forces applied to each node, and a 3*N* × 1 vector *U* represents the final displacements of each node (the final result of the FEM calculation). Mathematically, this is equivalent to solving for *U* via the following equation,

$$\begin{array}{c} K\cdot U=F. \end{array}$$ (5)

However, solving (*U* = *K*^−1^ ⋅ *F*) directly is computationally infeasible; iterative algorithms such as the biconjugate gradient stabilized method algorithm [22] are employed to estimate *U* by solving,

$$\begin{array}{c} U=\arg\min_{U}∥F-K\cdot U{∥}_{2}. \end{array}$$ (6)

*U* represents the displacement of nodes in the FEM (in mm).

To determine the displacement of each voxel in the image scene (*c* ∈ *C*), the nodes surrounding *c*, *S*_*c*_ ⊂ {1, …, *N*}, are defined by the corners of the FEM element containing *c*. The transformation of *c* is defined as an interpolation of nodal displacements,

$$\begin{array}{c} T\left(c\right)=c+\frac{\sum_{n\in S_{c}}∥c-v_{n}{∥}_{2}\cdot u_{n}}{\sum_{n\in S_{c}}∥c-v_{n}{∥}_{2}}, \end{array}$$ (7)

where **u**_*n*_ denotes the displacement of node *n* from the FEM result *U*. The transformed MRI T(C) is therefore defined as the conjunction of Eqs (1) and (7).

### Modeling Changes from Surrounding Tissue ${\hat{T}}_{2}$ Using an FEM

Even after taking into account patient motion and position within the MRI between visits (defined by ${\hat{T}}_{1}$), changes in tissues surrounding the prostate, such as the bladder and rectum, can cause deformations to the gland. To model how the bladder and rectum deform, an FEM is created by defining forces at the surface of these structures. The bladder and rectum filling and its effect on the prostate is modeled by an FEM, which is used to simulate how the MR image will deform due to these external forces. The direction and magnitude of the forces are defined by deforming the bladder and rectum on ${\hat{T}}_{1}\left(I_{Post}\right)$ towards the bladder and rectum on *I*_*Pre*_. The FEM calculates the deformation for the entire image given the forces at the surface of the bladder and rectum. These deformations are inverted and applied to the prostate. This has the effect of removing the deformations due to the bladder and rectum, and as such leaves only the treatment induced deformations remaining. This allows one to systematically and quantitatively track the shape-based changes due primarily to the treatment over time. The FEM based deformations (from the bladder and rectum on post- to pre-treatment) is denoted as,

$$\begin{array}{c} {\hat{T}}_{2}=FEM_{BR}\left({\hat{T}}_{1}\left(I_{Post}\right),I_{Pre}\right), \end{array}$$ (8)

where *FEM*_*BR*_(*a*, *b*) represents the FEM-induced deformations due to deforming the bladder and rectum from *a* to *b*.

### Focal Laser Ablation Induced Prostate Deformations *T*_3_

${\hat{T}}_{2}\left({\hat{T}}_{1}\left(I_{Post}\right)\right)$ represents the post-treatment image with the deformations due to the bladder and rectum removed. To model the focal laser ablation induced changes to the prostate, a FEM of the prostate is generated, and the prostate on ${\hat{T}}_{2}\left({\hat{T}}_{1}\left(I_{Post}\right)\right)$ is deformed to best first the prostate on *I*_*Pre*_. This deformation is denoted as *T*_3_ and defined as,

$$\begin{array}{c} T_{3}=FEM_{P}\left(I_{Pre},{\hat{T}}_{2}\left({\hat{T}}_{1}\left(I_{Post}\right)\right)\right). \end{array}$$ (9)

where *FEM*_*P*_(*a*, *b*) represents the FEM-induced deformations due to the prostate deforming from *a* to *b*. *T*_3_ represents the shape-based changes due primarily to the focal laser ablation. *T*_3_(*I*_*Pre*_) and ${\hat{T}}_{2}\left({\hat{T}}_{1}\left(I_{Post}\right)\right)$ represent spatially aligned pre-, post-treatment MRI respectively.

### Experimental Design

#### Data Description

A retrospective cohort of eight prostate cancer patients, scheduled for laser ablation, had T2-weighted MRI acquired both before and after the procedure. The cohort included patients from between 2008 and 2011, none of whom had any hormonal therapy. The time between pre-, post-treatment MRI scans ranged from 4–7 months. The data were anonymized and the study was approved by the Western Institutional Review Board (WIRB). In each study, the T2-weighted MRI was acquired using a 3.0 Tesla MRI scanner without an endorectal coil. The image sizes were approximately 140 × 140 × 140 mm, and the voxel sizes ranged from 0.27 × 0.27 × 2.2 mm/voxel to 0.54 × 0.54 × 3.0 mm/voxel.

#### Implementation Details

The algorithm was implemented in C++, based on the ITK framework [23], and compiled with gcc 4.8. Brock et al. [8] used a Young’s modulus of 21 kilopascals (kPa) for the prostate, and Chi et al. [11] claimed that normal prostate tissue has a Young’s modulus of 40-80 kPa, benign hypertrophic prostate tissue has a value of 30-50 kPa, and cancerous prostate tissue has a value of 80-120 kPa. Based on the preceding justifications for various ranges of Young’s modulus, in this study, we chose to use a Young’s modulus of 30 kPa for soft tissue, and found no significant differences in results from using slightly different values of Young’s modulus. In addition, in this work we chose use hexahedron elements for the FEM calculations as in [12, 21], and as such, $S_{c}\in R^{8}$. As in any biomechanical analysis, a tradeoff was made between computational complexity and accuracy of such a model. As such, we used the smallest element size possible (approximately 1mm x 1mm x 1mm) such that the experiments ran in under 1 hour.

#### *E*_1_: Applying Synthetic Deformations to Test FEM Inverse Accuracy

${\hat{T}}_{2}$ aims to remove the deformations on the prostate due to surrounding tissues. In this experiment, *T*_2_ is synthetically generated (defined as ${\tilde{T}}_{2}$) in order to quantify the accuracy of the inversion. If ${\hat{T}}_{2}$ perfectly recovered the deformations due to the bladder and rectum, then ${\hat{T}}_{2}={\left({\tilde{T}}_{2}\right)}^{-1}$

**Fig 2:**
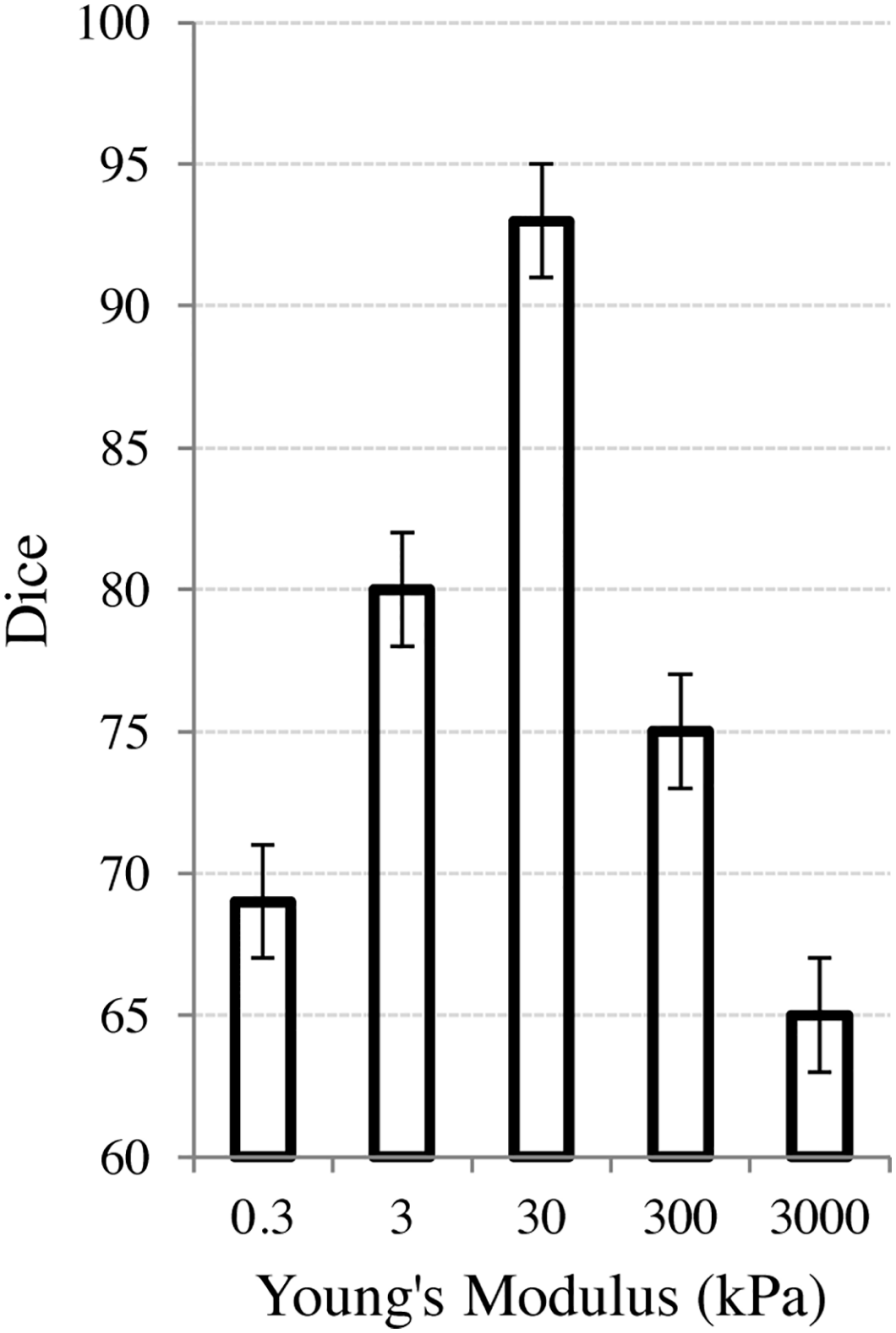
Effect of physical parameters on the FEM used to compensate for the bladder/rectum motion. The X-axis represents the Young’s modulus (in log-scale). The Y-axis represents the Dice similarity coefficient between the pre-treatment, undeformed prostate ($C_{Pre}^{P}$) and the prostate after inducing, and then removing, a synthetic simulation of the bladder and rectum filling (${\hat{T}}_{2}\left({\tilde{C}}_{Post}^{P}\right)$).

An FEM model of the bladder and rectum was created for the pre-treatment image on one study *I*_*Pre*_, and known forces at the surface were induced to generate a synthetic transformation ${\tilde{T}}_{2}$. The forces were chosen to deform the pre-treatment bladder and rectum towards the post-treatment bladder and rectum for the same study. This yields a synthetic post-treatment ${\tilde{I}}_{Post}$. We denote as $C_{Pre}^{P}$, the voxels contained within the prostate on the pre-treatment MRI scan. ${\tilde{C}}_{Post}^{P}={\tilde{T}}_{2}\left(C_{Pre}^{P}\right)$ represents the synthetically deformed post-treatment voxels. The Dice similarity coefficient [24] between $C_{Pre}^{P}$ and ${\hat{T}}_{2}\left({\tilde{C}}_{Post}^{P}\right)$ was used to determine the accuracy of the inversion, where a Dice of 100% indicates ${\hat{T}}_{2}={\left({\tilde{T}}_{2}\right)}^{-1}$. An analysis of the choice of Young’s modulus and its effects on the removal of the synthetic deformation is shown in Fig 2. The range of the log-scaled x-axis was from.3 kPa to 3000 kPa to highlight at what values of Young’s modulus, the accuracy significantly decreased. In the more typical biomechanical range of approximately 20-120 kPa, the resulting accuracy of the final Dice calculations were all greater than 92% in terms of accuracy.

#### *E*_2_: Using FEM to Quantify Changes Post-Treatment

For each step in the registration process, one image is fixed as the reference, and another image is considered the moving image. The first two steps bring *I*_*Post*_ into the frame of reference of *I*_*Pre*_, after which the focal laser ablation induced changes to the prostate are calculated to deform the pre-treatment MRI onto the post-treatment MRI. The result of this operation is a model of the prostate in which the external deformations have been removed., and in which the remaining shape-based changes are likely due primarily to the effects of ablation. The final deformation, *T*_3_ therefore represents the shape-based changes in the prostate due primarily to the focal laser ablation treatment. This is compared to the location of treatment in order to determine the magnitude of treatment-induced shape-based changes at the site of ablation. Such changes to the prostate shape at the site of ablation can be used to quantitatively track treatment response over time.

## Results

### *E*_1_: Applying Synthetic Deformations to Test Accuracy of Removing Effects of Bladder and Rectum Filling on Prostate

The synthetic experiments outlined in Section 3 resulted in a mean Dice score of 93% ± 2%, suggesting that the FEM was able to accurately recover the bladder and rectum deformations. Fig 2 shows the effects of the Young’s modulus on the ability of the FEM to recover the deformations. The induced synthetic deformation (prior to using the FEM to recover the deformation) started at a Dice value of 65%. As the Young’s modulus veered away from the soft-tissue value of 30 kPa, the deformation was less able to be recovered.

**Fig 3:**
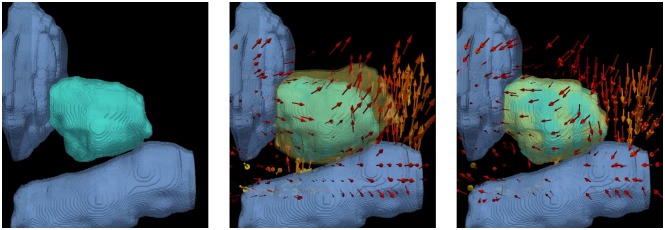
Illustration of the ablation related effects on the prostate, bladder, and rectum modeled by the FEM. ((a)) represents the bladder and rectum in pale blue, and prostate in teal. ((b)) represents the synthetic deformation *T*_2_. The deformed prostate is shown in yellow, and the arrows in ((b)) represent the direction of the transform (mostly due to the rectum). ((c)) represents the result of the recovered deformation ${\hat{T}}_{2}$. The deformed prostate is shown in semi-transparent yellow in ((b)) and ((c)). The high level of overlap between the semi-transparent yellow prostate and the teal prostate in ((c)) shows the accuracy of removing the simulated effects of the bladder and rectum motion.

Fig 3 shows the qualitative results of *T*_2_ in Fig 3(b) and the recovered deformation ${\hat{T}}_{2}$ in Fig 3(c). In this case, the change in the rectum (pale blue, below) was the primary driving force in pushing the prostate (teal) upwards, near the apex. The pulling effect caused by bringing the rectum back to its original position caused the inverse deformation in the prostate, yielding a high overlap with the undeformed, pre-treatment prostate in Fig 3(c).

### *E*_2_: Using FEM to Quantify Changes Post-Treatment

#### Volume Changes

**Fig 4:**
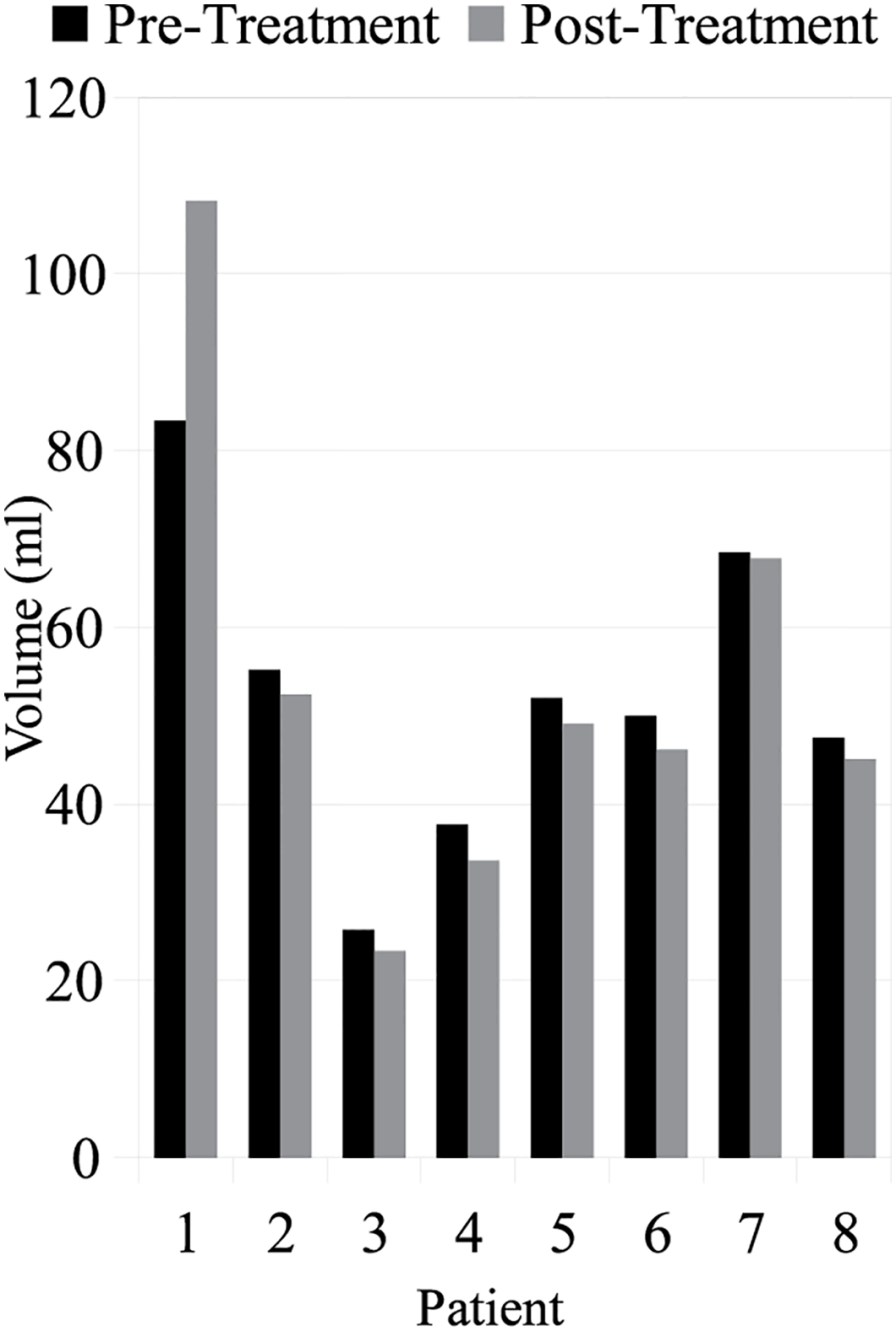
The prostate volume across all eight patients both before and after treatment. The median change was a 5% decrease in volume following focal laser ablation treatment.

Fig 4 shows the prostate volume before and after focal laser ablation treatment. The median pre-treatment volume was 51.0 ml and the median post-treatment volume was 47.7 ml, a decrease of 5.1%. Since the effects of patient positioning and nearby organ changes were removed prior to calculating the change in volume, this decrease suggests that the treatment itself caused a shrinking effect within the prostate.

#### Shape-Based Changes

**Fig 5:**
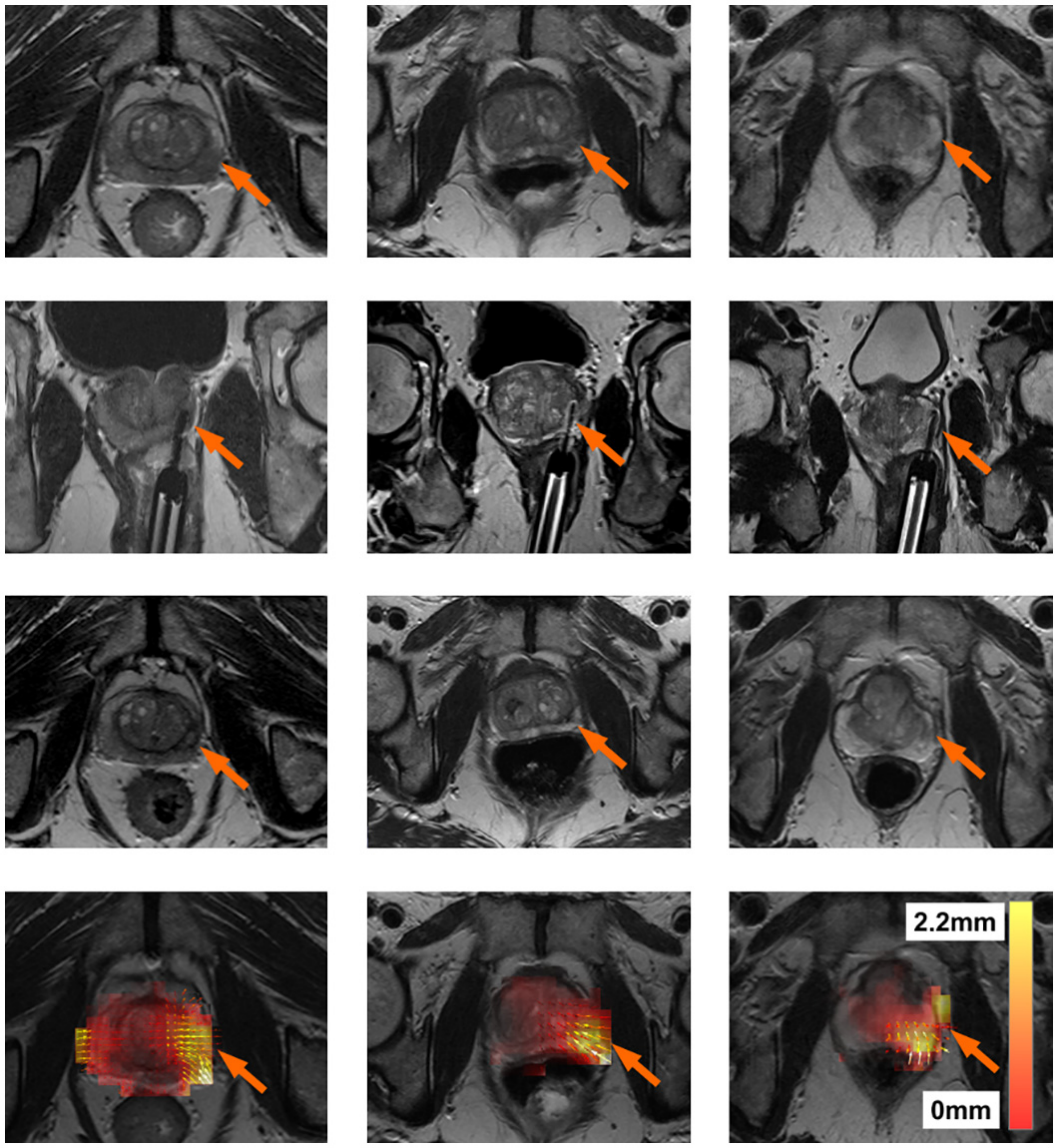
Results for three patients (one per column). The first row represents the pre-treament MRI scan (*I*_*Pre*_). The second row represents the location of the laser during treatment. The third row represents the post-treament MRI scan (*I*_*Post*_). The fourth row represents a heat map of the ablation induced deformations *T*_3_. White represents regions of large deformations (2.2 mm), while transparent red represents regions of small deformations (0 mm). Small arrows represent the direction of the deformation (in all cases pointing towards the centroid of the prostate) after removing deformations due to patient alignment (*T*_1_) and surrounding tissues (*T*_2_). It can be seen that in all patients, the areas with the the largest deformations correspond to the focal laser ablation sites.

Fig 5 shows the registration result for three patients in order to determine where the shape-based changes in the prostate occurred. Each patient is represented by a column. The first row shows *I*_*Pre*_, the second row an image of the ablation needle during treatment, and the third row shows *I*_*Post*_. The slight change in volume in the prostate is visible on *I*_*Post*_. The registration result *T*_3_ is shown in the fourth row. The arrows represent the direction of the shape-based changes, and in all cases they point inwards towards the center of the prostate close to the site of ablation. In addition, the deformation heatmap shows the magnitude of shape-based changes (∥*T*_3_(*c*) − *c*∥_2_), where red represents a small change and white represents a large change. These results show that a slight decrease in volume of the prostate occurred at the site of ablation, suggesting that the focal laser ablation induced necrosis caused a change in prostate morphology and shape.

#### Post-Ablation MRI Changes

**Fig 6:**
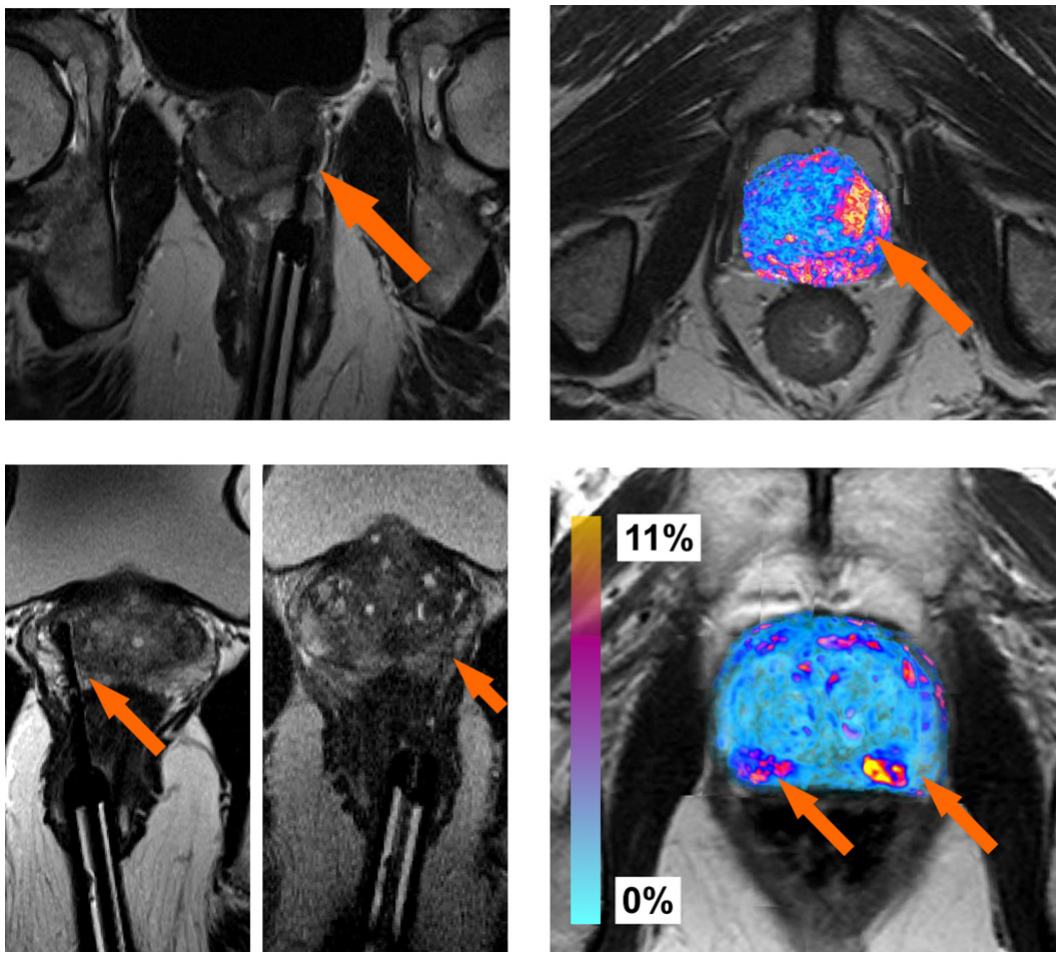
Illustration of the focal laser ablation needle locations on T2-w MRI during the procedure for two different patients ((a), (c)). After registration, the difference between the MRI intensity values (as a relative percent change) are shown as colored values in ((b)) and 3D ((d)), where cool blue colors represent regions corresponding to small differences (0%), while hot red colors represent regions of large differences (11%) in MRI intensity values. Most of the hot colors are correlated with the needle locations (shown as orange arrows).

Fig 6(a) and 6(b) show MRI scans both during and after the laser treatment, respectively. The laser needle during the procedure at the site of ablation is clearly visible in Fig 6(a). The changes in MRI intensity values following the FEM based registration are shown as a colored heatmap in Fig 6(d). Hot colors represent areas of large changes in MRI intensity values and cooler colors represent areas of small changes. The same representations are shown for a second patient in Fig 6(c) and 6(d). This particular patient had two sites of ablation, both shown by the MRI images during treatment in Fig 6(c).

## Discussion

### Volume Changes

With regards to the treatment induced volume changes, in most patients, the volume of the prostate decreased following focal laser ablation treatment. However, in patient #1 (the patient with the largest prostate), the volume actually increased following treatment. While the imaging scan and treatment options for patient #1 were no different than the other patients, the one noticeable difference was that patient #1 had the largest pre-treatment prostate volume. While this increased volume could have been due to a number of different factors (e.g. prostatic hypertrophy), in future studies this confounding factor will have to be controlled for to assess changes solely due to the treatment modality. One way of doing this is via an atlas based approach, such as was presented in [25], where all the prostates within a population were mapped into a single canonical representation which allows for size normalization, so that additional changes can then be attributed primarily to the treatment.

### Shape-Based Changes

With regards to the shape-based changes, all three patients shown in Fig 5 had significant shape-based changes at the ablation site. Fig 5(j), 5(k), and 5(l) all show significant shape-based changes in the bottom right corner. Fig 5(d), 5(e), and 5(f) all show that the ablation sites (seen via the laser needle) are directly related to the location of the aforementioned shape-based changes.

### Post-Ablation MRI Changes

With regards to the functional changes on MRI, the regions corresponding to the top of the prostate in Fig 6(b) and 6(d), show slight changes in intensity values. This was caused by a very slight misalignment of the prostate boundaries within this region. However, at the two focal laser ablation sites seen in Fig 6(c), there is significant necrosis following treatment, showing up as hot colors in Fig 6(d). These results are also apparent in the difference map from a second patient, shown in Fig 6(b), who only had one ablation site, shown in Fig 6(a). In both cases, there were large changes in the MRI parameter values at the ablation sites. While there are regions of MRI intensity value changes pre-, post-treatment due to misalignment, most of the changes in MRI intensity values occurred near the sites of ablation. We believe that these changes in MRI intensity values could correlate to functional changes within the prostate, which may help quantitatively track patients (and associated treatment efficacy) over time. The sites of significant changes outside of the ablated zone are likely due to either (a) slight misalignment of the pre-, post-treatment MRI scans, or (b) changes within the tissue itself due to the large time interval between scans. Other contributory factors could include diet and disease progression which could have occurred within that timespan between scans.

### Sources of Variability

There are several potential sources of variability in this study listed below.

- **# of Patients**: This preliminary study included 8 patients. To evaluate the generalizability of this method in order to quantitatively determine (and track) treatment-related changes over time, our biomechanical model will in the future need to be evaluated on a much larger cohort of patients.
- **Time Between Scans**: The time between the pre-, post-treatment scans can introduce changes in the prostate due to non-treatment related reasons. Our attempt to remove the effects of the bladder and rectum on the shape of the prostate was used to mitigate this effect as much as possible. Yet internal morphologic changes within the gland unrelated to the treatment or implicitly due to the treatment (e.g. vascular changes, disease recurrence) could potentially result in “false positive” zones, in which the MRI image’s intensity values could have changed between scans. However, the fact that the majority of the changes were located near the site of ablation suggests that these changes were largely and primarily due to the treatment itself.
- **Uniform Young’s Modulus**: In this study, the final results did not vary significantly if we varied the Young’s modulus throughout the gland while using the FEM to model the organ, compared to using a homogeneous Young’s modulus value. However it is entirely possible that the use of a heterogeneous Young’s modulus to model the deformation of the entire prostate could result in slightly different deformation results compared to what we observed in this study.
- **Segmentation Accuracy**: The use of manual segmentations in this study was meant to reduce the potential for the segmentation accuracy being a source of error in the FEM accuracy. However, we recognize that different observers may segment the gland slightly differently, and that the use of an automated segmentation algorithm as a pre-preprocessing step (prior to generating the FEM) could potentially be a source of variability. Yet it is important to note that most sites of ablation in this study were in the midgland, which we previously found [26] to have higher segmentation accuracy and lower variability than in the apex or base.

## Conclusion

Focal laser ablation treatment aims to destroy cancerous cells with highly focused laser, in order to cause necrosis to the affected tissue. It combines the benefits of aggressive therapy such as radiation treatments (the ability to destroy cancers cells) without the harmful side effects (due to its localization). Quantifying treatment related changes (both shape-based as well as functional) to the prostate can be used to systematically track a patient over time, as well as potentially develop predictive models for long term patient outcome. However, to quantify these changes, the pre- and post-treatment MR images must first be spatially aligned via image registration. Challenges of such a registration technique arise from the significant changes to gland morphology following treatment due to (1) patient alignment, (2) changes due to surrounding organs such as the bladder and rectum, and (3) changes due primarily to the focal laser ablation itself.

In order to isolate the treatment induced shape-based changes, the changes from (1) and (2) were first modeled and removed. Then, a finite element model (FEM) was used to determine the ablation induced changes to the prostate. This resulted in (a) ablation induced shape-based changes to the prostate, and (b) spatially aligned pre-, post-treatment imagery. To the best of our knowledge, our approach is the first attempt to use a FEM to isolate the treatment-related shape-based changes in the prostate.

This methodology was applied to eight patients in order to quantify (1) changes in the prostate volume following treatment, (2) locations of significant shape-based changes within the prostate due primarily to the treatment, and (3) locations of changes in MRI intensity values following registration of pre-, post-treatment imagery. The patients used in this cohort only had focal laser ablation treatment, removing hormonal therapy as a possible confounding factor when quantitatively tracking patients over time. Our results suggested that focal laser ablation usually causes a decrease in prostate volume, specifically located at the site of ablation. In addition, changes in MRI intensity values, which may be correlated to functional changes within the prostate, typically appear occur at the site of ablation.

This quantification of shape-based changes of the prostate could pave the way for determining possible correlations between shape-based changes in the prostate and treatment response. In addition, this approach defines a framework for isolating treatment-related shape-based changes in other domains such as liver and lung treatment.

In this work we present results which suggest that the focal laser ablation treatment causes a minor decrease in prostate volume, focused specifically at the site of ablation. In addition, after spatially aligning the images, changes to MRI intensity values are clearly visible at the site of ablation. Both these results lend themselves to quantifying the degree of ablation induced changes to the prostate, which can be used to track a patient’s treatment response to prostate cancer therapy over time.

One limitation of this study is that additional confounding factors may contribute to prostate deformation such as blood flow or concomitant therapies. We assume that these effects are minimal compared to the effects from the bladder and rectum. A second limitation of this study is the dependency on the accuracy of the organs’ segmentations, which future work will aim to explore.

In this paper we reported preliminary results of a novel method for fusing pre-, post-treatment MRI in order to quantitatively investigate changes in the prostate. Future work will entail evaluating this method on a larger cohort of patients, in order to determine if such a method could be useful for tracking treatment efficacy over time.
